# Corrigendum: Eupafolin Suppresses Esophagus Cancer Growth by Targeting T-LAK Cell-Originated Protein Kinase Protein Kinase

**DOI:** 10.3389/fphar.2020.01178

**Published:** 2020-08-14

**Authors:** Xiaoming Fan, Junyan Tao, Xin Cai, Mangaladoss Fredimoses, Junzi Wu, Zhihui Jiang, Kunpeng Zhang, Shude Li

**Affiliations:** ^1^ Henan Joint International Research Laboratory of Veterinary Biologics Research and Application, Anyang Institute of Technology, Anyang, China; ^2^ Institute of Environmental Safety and Human Health, Wenzhou Medical University, Wenzhou, China; ^3^ Laboratory of Natural Product Extraction, China-US (Henan) Hormel Cancer Institute, Zhengzhou, China; ^4^ College of Basic Medical, Yunnan University of Chinese Medicine, Kunming, China; ^5^ Department of Biochemistry and Molecular Biology, School of Basic Medicine, Kunming Medical University, Kunming, China; ^6^ Yunnan Province Key Laboratory for Nutrition and Food Safety in Universities, Kunming, Yunnan, China

**Keywords:** eupafolin, esophagus cancer, TOPK, inhibitor, Ay Tsao

**Xin Cai** was not included as an author, and **Xiaoying Zhang** was incorrectly included as an author in the published article. The corrected Author Contributions Statement appears below.

## Author Contributions

XF designed research, performed research and wrote the paper; JT analyzed the data; MF extracted Eupafolin from Ay Tsao; JW expressed Histone H3 protein; XC, ZJ performed animal research andanalyzed data; SL, KZ designed research and analyzed data.

Furthermore, there was a mistake in **Figure 1D** as published. The TOPK western picture has an extra strip. The corrected **Figure 1** appears below.

**Figure 1:**
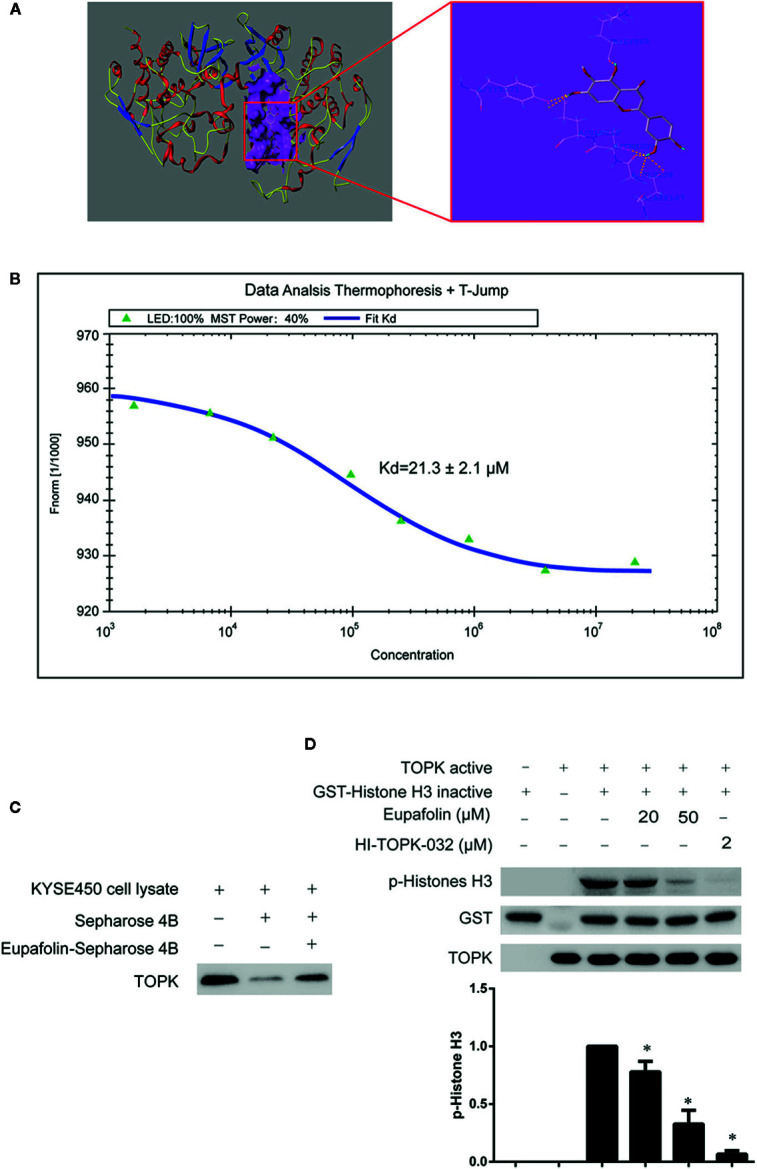
Eupafolin binds with TOPK and suppresses TOPK activity in vitro. **(A)** The docking model of eupafolin and TOPK. **(B)** Measurement of affinity between TOPK and eupafolin by MST in standard treated capillaries, and the resulting binding curve was shown. From the resulting binding curve, Kd of 21.3 ± 2.1 is calculated. **(C)** Eupafolin binds directly with TOPK. Sepharose 4B was used for binding and pull-down assay as described in section “Materials and methods.” Lane 1 is input control (TOPK protein standard); lane 2 is the negative control, indicating there is no binding between TOPK and beads alone; and, lane 3 indicates that TOPK binds with eupafolin-Sepharose 4B beads. **(D)** Eupafolin inhibits TOPK activity in vitro. The inhibitory effect of eupafolin on TOPK was determined by an in vitro kinase assay. An inactive GST-histone H3 protein was used as the substrate with active TOPK and 100 μM ATP in the reaction buffer. Protein were resolved by 10% SDS-PAGE gel and detected by Western blot. Histogram statistics is the expression of the p-histone H3 in the first line. Data are representatives of results from triplicate experiments. *Significant compared with lane 3 alone, P < 0.05.

The authors apologize for these errors and state that this does not change the scientific conclusions of the article in any way. The original article has been updated.

